# Health provider perspectives on the implementation of the same-day-ART initiation policy in the Gauteng province of South Africa

**DOI:** 10.1186/s12961-020-00673-y

**Published:** 2021-01-06

**Authors:** Dorina Onoya, Idah Mokhele, Tembeka Sineke, Bulelwa Mngoma, Aneesa Moolla, Marnie Vujovic, Jacob Bor, Jonas Langa, Matthew P. Fox

**Affiliations:** 1 Health Economics and Epidemiology Research Office, Department of Internal Medicine, School of Clinical Medicine, Faculty of Health Sciences, University of the Witwatersrand, Johannesburg, South Africa; 2 Right to Care, Johannesburg, South Africa; 3 Departments of Global Health, Boston University School of Public Health, Boston, MA United States of America; 4 Departments of Epidemiology, Boston University School of Public Health, Boston, MA United States of America

**Keywords:** HIV, ART attrition, Universal-test-and-treat, Same-day ART, Health provider

## Abstract

**Background:**

In September 2016, South Africa (SA) began implementing the universal-test-and-treat (UTT) policy in hopes of attaining the UNAIDS 90-90-90 targets by 2020. The SA National Department of Health provided a further directive to initiate antiretroviral therapy (ART) on the day of HIV diagnosis in September 2017. We conducted a qualitative study to determine the progress in implementing UTT and examine health providers' perspectives on the implementation of the same-day initiation (SDI) policy, six months after the policy change.

**Methods:**

We conducted in-depth interviews with three professional nurses, and four HIV lay counsellors of five primary health clinics in the Gauteng province, between October and December 2017. In September 2018, we also conducted a focus group discussion with ten professional nurses/clinic managers from ten clinic facilities. The interviews and focus groups covered the adoption and implementation of UTT and SDI policies. Interviews were conducted in English, Sotho or Zulu and audio-recorded with participant consent. Audio-recordings were transcribed verbatim, translated to English and analysed thematically using NVivo 11.

**Results:**

The data indicates inconsistencies across facilities and incongruities between counsellor and nursing provider perspectives regarding the SDI policy implementation. While nurses highlighted the clinical benefits of early ART initiation, they expressed concerns that immediate ART may be overwhelming for some patients, who may be unprepared and likely to disengage from care soon after the initial acceptance of ART. Accordingly, the SDI implementation was slow due to limited patient demand, provider ambivalence to the policy implementations, as well as challenges with infrastructure and human resources. The process for assessing patient readiness was poorly defined by health providers across facilities, inconsistent and counsellor dependent. Providers were also unclear on how to ensure that patients who defer treatment return for ongoing counselling.

**Conclusions:**

Our results highlight important gaps in the drive to achieve the ART initiation target and demonstrate the need for further engagement with health care providers around the implementation of same-day ART initiation, particularly with regards to infrastructural/capacity needs and the management of patient readiness for lifelong ART on the day of HIV diagnosis. Additionally, there is a need for improved promotion of the SDI provision both in health care settings and in media communications to increase patient demand for early and lifelong ART.

## Background

Sub-Saharan Africa remains the region worst affected by the HIV epidemic, accounting for more than two-thirds of the global HIV burden [1]. Despite this, the region has seen substantial gains in the fight against HIV in recent years with the expansion of antiretroviral therapy (ART) eligibility, and subsequent adoption of the World Health Organisation (WHO) recommended universal-test-and-treat (UTT) policy [2–4]. However, many health systems across Sub-Saharan Africa remain weak, under-resourced and overburdened [5–7]. Furthermore, many countries in the region faced challenges in meeting UNAIDS 90-90-90 HIV targets and fully realising the benefits of the UTT policy due to persistent health system deficiencies [1, 7, 8].

South Africa (SA) bears the largest HIV burden in the region, with nearly eight million individuals living with HIV, and over four and a half million of these receiving ART in 2019 [9, 10]. However, despite considerable efforts to scale-up access to treatment, an additional three million individuals need to start ART to reach 95% of HIV diagnosed patient on ART by 2030 [4, 11, 12].

Evidence of the benefits of ART are well-documented [13–15]. As a result, the SA government adopted the UTT strategy in 2016 and the ART same-day initiation (SDI) policy in 2017 [13, 15–19]. While the UTT policy removes clinical barriers to ART initiation, the SDI policy aims to reduce the time from HIV diagnosis to ART start to one visit. The SDI policy makes ART initiation logistically easier for patients and can further reduce patient losses in the pre-ART phase of care. However, in SA and other low-and-middle-income countries (LMIC), the implementation of the UTT and SDI policies were not accompanied by expanded human resources or infrastructural capacity [5–7]. The resulting demand for ART can potentially increase the pressure on already burdened public health services, and further compromised the quality of care [16, 20, 21].

Therefore, it is, essential to understand how public sector healthcare providers have received these policies and managed their enactment to identify gaps and devise solutions to maximise and sustain benefits. In this study, we aimed to explore progress towards UTT policy assimilation and examine primary health care providers' perspectives on the implementation of same-day ART initiation after the policy adoption.

## Methods

### Study setting and sites

The study was conducted at eleven primary health clinics in Johannesburg, South Africa between October 2017 and September 2018.

### Participant characteristics

A total of fifteen health providers from eleven clinics participated in the study (Table 1). Eleven of the health providers were professional nurses, and eight of these were also facility managers. Four lay HIV counsellors were also interviewed from four different clinics.

**Table 1:** Overview of study participants

Type of provider	Data collection activity		Total
	Key informant interviews	Focus group discussion	
Professional nurse (PN)	–	3	3
Clinic manager/professional nurse	3	7	10^a^
Lay HIV counsellor (LHC)	4	–	4
Total	7	10	15^a^

^a^Two clinic manager/professional nurse took part in both KI interviews and the FGD

### Key informant interview procedures

We conducted key informant interviews with three professional nurses and four lay HIV counselling and testing counsellors at four of the study sites (Table 1). Interviews lasted approximately 45 min and were conducted in a private space within the clinic by a trained interviewer. The interview guide explored processes involved in HIV testing services, ART initiation, patient management and follow-up under the UTT and SDI policies, and also explored providers' understanding and attitudes towards the policy changes and implementation processes. Interviews were conducted in English, Sotho or Zulu, and were audio-recorded. Audio recordings were transcribed verbatim and translated to English for analysis.

### Focus group procedures

Additional data was collected via a focus group discussion with professional nurses/clinic managers from ten primary health clinic facilities, covering topics related to the management of the UTT and SDI implementation processes. The discussion lasted approximately 60 min and was conducted in a venue provided by the researchers, outside the health providers' workplaces. Discussions were conducted in English and were also audio recorded. All audio recordings were transcribed verbatim.

### Data analysis

All transcripts were analysed thematically using NVIVO software which facilitated data management and coding. Transcripts were read and coded by three research team members individually. Initial themes were drawn from topics covered in the interview guide. Major trends and cross-cutting themes were identified and then refined over several meetings. Any coder variation identified was resolved through discussion and consensus from all research team members.

All participants provided written informed consent before all data collection procedures. Confidentiality and anonymity were safeguarded by removing all identifiers, including participants' names and names of facilities from the data. This study was approved by the Human Research Ethics Committee (Medical) of the University of the Witwatersrand (Wits HREC M1704122 and M170579).

## Results

**Fig. 1:**
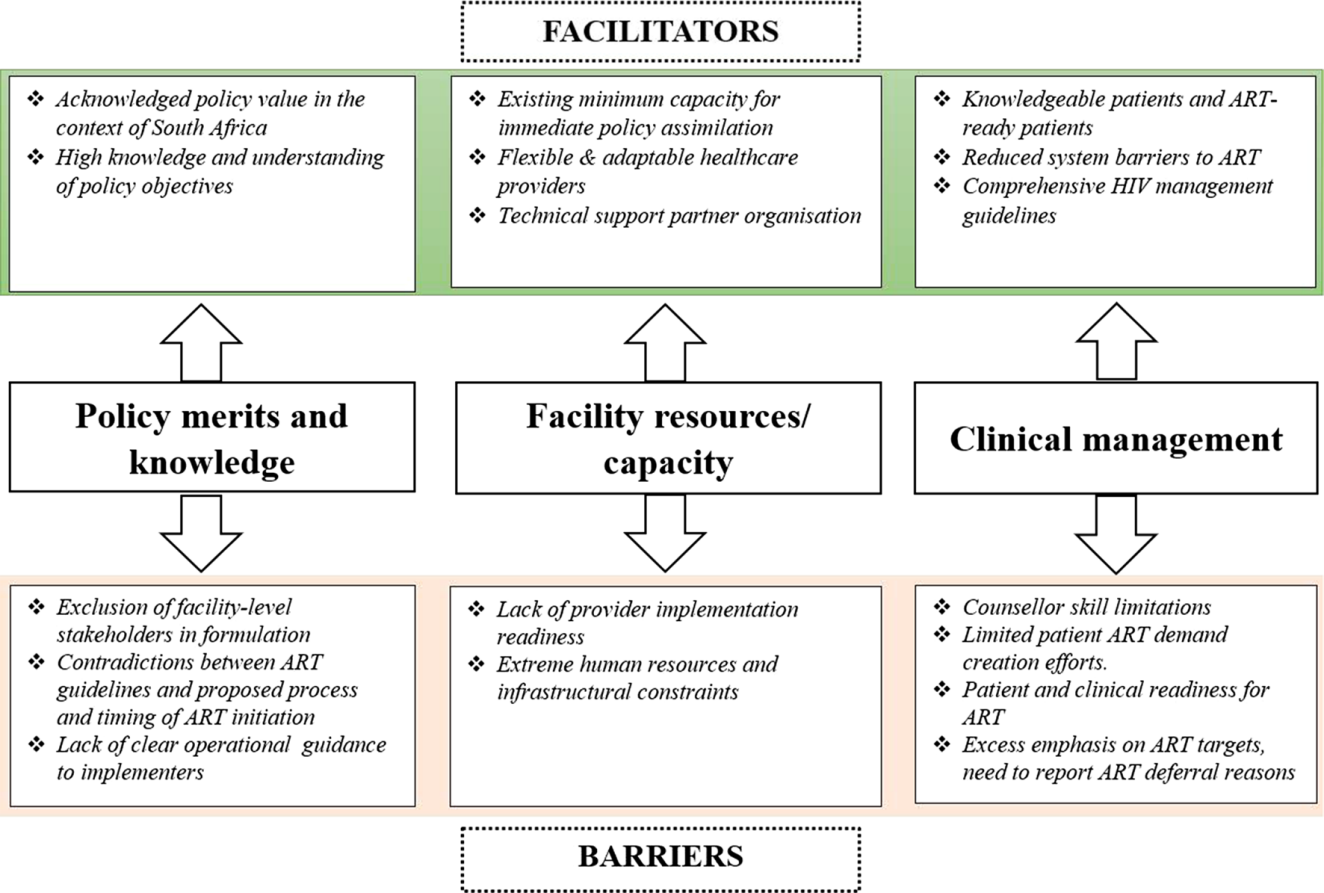
Summary of barriers and facilitators to the same-day ART policy implementation at primary healthcare facilities in Johannesburg, South Africa

Primary health care providers highlighted several factors affecting policy implementation at the healthcare management level, clinic and provider levels, and patient-level which are summarised below, and in Fig. 1, with supporting quotations presented in Table 2.

**Table 2:** Policy merit and healthcare worker knowledge related facilitators and barriers to UTT policy and same-day ART initiation implementation

Barriers and facilitators	Illustrative quotes
Facilitators	
Policy value in the context of South Africa	"…like I say, the aim [of the policy changes] is to put everyone on [treatment], everybody who is positive so that there must not be any transmission”—PN
Knowledge of policy objectives	“I like it [UTT] because some people they say we are going to see, like if the CD4 count is less than 500 they start to initiate. But if it’s more than 500 they don’t initiate that person, and that person they get sick. They get sick even though their CD4 count is high. So, that is not right. To start initiate the person who is very sick, because the side effects worse in that person, it is going to be worse for the person. So, it’s better to test and treat while they’re still healthy”—LHC
Barriers	
Exclusion of facility-level stakeholders in policy formulation	“For me, it would have worked very better if before a policy is being designed for the facility, even before the level of the operations manager, they call their workshop. So, that they’ve got buy-in and understanding. Because sometimes you will try to explain a policy that you, yourself don’t understand…”—PN
Perceived contradictions between ART guidelines and proposed process for ART initiation	“I think again patients’ readiness, there’s subjective readiness and there’s objective readiness. My being readiness to [initiate] doesn’t necessarily meaning I qualify for you to initiate, we still have those patients that we will need to fast-track, the guidelines remains the same, antenatal, TB patients. I might be ready to take but then we have to exclude other conditions. You find out I’m ready, I’m saying yes, I’m ready, probably because… when you get deeper into literature, you know those patients, there’s certain categories of patients that you know such patients are better ready compared to these patients.:—PN
Lack of clear operational guidance to implementers	“Like when you attended meetings… they kept saying you can initiate patients immediately. But, you know the clinicians [nurses] were not that confident because they were saying they don’t have anything written in black and white that guides them.”—PN “…what I noticed especially with the nurses at our clinic is that they were also resistant to the very UTT because it was not implemented like when NIMART implemented, people went for training, you know, expensive training. But with UTT, just a memo came and said from now on you can do this, and you don’t have to wait for this, and you don’t have to wait for that.”—PN

### Universal-test-and-treat and same-day ART initiation policy knowledge

#### Acknowledged policy value in the context of South Africa

All primary care providers were aware of the policy changes and understood the significance of early ART initiation to improve clinical outcomes but also to prevent transmission to HIV negative partners. However, they expressed concerns around the implementation of the same-day ART initiation directive, given the prevailing clinic resource and their perception of patient psychological needs and social circumstances.

“…like I say, the aim [of the policy changes] is to put everyone on [treatment], everybody who is positive so that there must not be any transmission”—PN

#### Lack of clear operational guidance to implementers

Primary healthcare providers highlighted the lack of detailed guidance around the new policy as a critical barrier to the smooth transition to the new policies. Facility managers noted that staff nurses were hesitant to implement immediate ART because they perceived contradictions between the previous (detailed) ART guidelines and the proposed process for ART initiation on the day of HIV diagnosis. As a result, most facility managers struggled to motivate their staff to implement SDI when the directive was first received. Many still stressed the need to ensure that patients were clinically ready for ART before initiation. They were concerned that initiating patients on ART without baseline safety laboratory test results was contrary to their training and prevailing HIV treatment guidelines. Some referenced the extensive training that was provided to nurses in preparation for the implementation of the Nurse Initiated Management of ART (NIMART) as a good example of how the SDI directive should have been introduced [22]. Training for the NIMART program contained detailed process guidelines creating self-efficacy in nurses who were then tasked with ART initiation and management at primary health care clinics [22–24].

“Like when you attended meetings… they kept saying you can initiate patients immediately. But, you know the clinicians [nurses] were not that confident because they were saying they don’t have anything written in black and white that guides them.”—PN.

“…what I noticed especially with the nurses at our clinic is that they were also resistant to the very UTT because it was not implemented like when NIMART implemented, people went for training, you know, expensive training. But with UTT, just a memo came and said from now on you can do this, and you don’t have to wait for this, and you don’t have to wait for that.”—PN.

#### Inclusion of facility-level stakeholders in policy formulation and implementation planning

Participants underlined the necessity of involving facility-based health providers in policy implementation planning. Primary care providers were familiar with clinic conditions and primarily responsible for the actual policy implementation.

“For me, it would have worked very better if before a policy is being designed for the facility, even before the level of the operations manager, they call their workshop. So, that they’ve got buy-in and understanding. Because sometimes you will try to explain a policy that you, yourself don’t understand…”—PN.

### Health system resources and capacity

#### Extreme human resources and infrastructural constraints

Primary care clinics have been tasked with HIV testing, ART initiation and HIV patient management since 2010 as part of efforts to expand patients' access to ART [23, 24]. The same-day ART initiation policy eliminates the pre-ART activities and now compresses all ART preparation sessions into one long initial (diagnosis) visit, limiting the total number of patients that could be seen per day. Human health resource shortages were cited as a critical barrier to policy implementation. Providers perceived current staffing capacity to be already strained, and that the same-day ART initiation policy would exacerbate the already overcrowded conditions at the clinics. Clinic managers also noted that the expectation to lead the implementation of the new policies in clinics that were initially designed and resourced to provide mainly primary (non-HIV) care services was challenging.

“… much as it’s [SDI] not a new service that we are implementing, […] even at the level of the clinician that time that the clinician takes to make a proper initiation […] and the fact that the resources were never altered. The very same clinicians that were doing other things is expected to do that. Now you are seated with the clinician and that counsellor who are compromised in terms of the quality and standard of care that they can provide, but expected on the other hand, to be implementing the policy itself”—PN.

“Because our clinic when it was formed, or built or whatever…I don’t know exactly the history but it wasn’t for all the services. Hence…It’s a very small clinic, and the service was just for family planning, EPI [expanded program on immunization] and but then it started increasing service…and then in June 2016 we started for the first to actually give treatment to HIV positive clients”—PN.

#### Adaptable healthcare providers and non-governmental organisations (NGO) partner dependency

Facility managers emphasised that their requests for additional human, infrastructural and material resources often remain unfulfilled. The resource limitations require some creativity in the use of clinic space (offering their administration offices or, in some cases, the emergency room to be used as consulting rooms), staff and clinic processes to manage high volumes of patients more effectively (Table 3).

**Table 3:** Health system resources and capacity related facilitators and barriers to UTT policy and same-day ART initiation implementation

Barriers and facilitators	Illustrative quotes
Facilitators	
Existing minimum capacity for immediate policy assimilation	“…much as [SDI] it’s not a new service that we are implementing, but if you’re going to do that [implementing SDI], even at the level of the clinician, that time that the clinician takes to make a proper initiation to the patient and the fact that the resources were never altered.”—PN
Flexible healthcare providers conditions	“So, with space issues, I think there was a time when we used to do that…when we used to use the emergency room and then you are busy with a client and then an emergency comes in and you have to go, where do you go?”—PN “Sometime I would have to vacate my office, and say okay finish whatever and then I’m just…you know…roaming around the clinic”—PN
Technical support partner organisation	“So, they were like, no we don’t have anything in black and white that covers us. But, fortunately [because of the NGO support partner], we had something. They gave us something and referred us to the UTT policy and then they gave us the flow chart, just to guide us to that if patients do this, at least we’ve got the flow chart…”—PN
Barriers	
Lack of provider implementation readiness	“…what I noticed especially with the nurses at our clinic is that they were also resistant to the very UTT because it was not implemented like when NIMART implemented, people went for training, you know, expensive training. But with UTT, just a memo came and said from now on you can do this, and you don’t have to wait for this, and you don’t have to wait for that.”—PN
Extreme human resources and infrastructural constraints	“Because our clinic when it was formed, or built or whatever…I don’t know exactly the history but it wasn’t for all the services. Hence…It’s a very small clinic, and the service was just for family planning, EPI and but then it started increasing service…and then in June 2016 we started for the first to actually give treatment to HIV positive clients”—PN “[…] the fact that the resources were never altered, the very same clinicians that were doing other things is expected to do that. Now you are seated with the clinician and that counsellor who are compromised in terms of the quality and standard of care that they can provide, but expected on the other hand, to be implementing the policy itself”—PN “Hence when we started I mentioned the duties of NGOs…how do they come…how do you say I’m going to support this person…how are you going to? Firstly, the infrastructure is absolutely wrong. You don’t even fit in there [physically], human resource-wise you can’t squeeze somebody there but you say that I’m going to support these people.”—PN

There was a heavy reliance on support partners to assist with the implementation of new policies. These included non-governmental organisations (NGOs) that provided technical support and sometimes temporary structures to address human resources and consultation space constraints. Often, these NGO partners are also relied on for training HIV counselling and testing staff. In most cases, NGO partners place additional counsellors or tracing officers in already overstretched clinics with sometimes unclear long-term added benefits. The support of NGO partners is particularly noted in the capturing and management of ART monitoring data.

Nonetheless, primary care providers believed that NGO partners' technical support efforts would have little impact on their clinics' ability to sustainably achieve their goals if resource and infrastructure shortfalls are not addressed. The majority of participants expressed an expectation that NGO partners should negotiate on behalf of facilities that they are contracted to support, considering their familiarity with the clinic resources needs and the partners' apparent access to higher-level health authorities and funding organisations.

“So, with space issues, I think there was a time when we used to do that…when we used to use the emergency room and then you are busy with a client and then an emergency comes in and you have to go, where do you go?”—PN.

“Hence when we started I mentioned the duties of NGOs…how do they come…how do you say I’m going to support this person…how are you going to? Firstly, the infrastructure is absolutely wrong. You don’t even fit in there [physically], human resource-wise you can’t squeeze somebody there but you say that I’m going to support these people.”—PN.

### Clinical management of HIV positive patients

#### Knowledgeable patients and ART-ready patients

Nurses highlighted that urban patients are increasingly knowledgeable about HIV and ART, thus reducing some of the ART readiness challenges. HIV and ART information campaigns lighten the health education burden of HIV counselling and testing counsellors who can focus on correcting myths. However, they expressed fears that knowledge about ART as a prevention intervention would reduce the emphasis on other HIV/STI prevention messages, particularly the use of barriers protection methods. Health providers also expressed concerns that some prevention messages (such as the availability of pre-exposure prophylaxis) may be suppressed because of cost and logistical considerations. Hence the need for trained counsellors to handle increasingly complex HIV counselling and testing requirements to prepare patients for rapid ART initiation and lifelong adherence to treatment.

“Today we live with people who are HIV positive. Although we may speak of people who are in a remote place…and you know…this person…you may give us cues relating to that. But for somebody who is living in CBDs and all that urban life, and all that stuff. They should know about [HIV], even at workplace, I think that’s something…on TVs we talk about HIV…you know…or related things.”—PN.

“It doesn’t necessarily mean transmission will only be stopped by treatment. I think these days were just talking about treatment, treatment—we don’t talk about condoms anymore, we don’t talk about healthy lifestyle anymore. We just want treatment, treatment…”—PN.

#### Counsellor skill limitations

Health providers were nearly unanimous in their call for additional support and training for lay counsellors because of their critical role patients' engagement in the HIV care cascade. Additional skills training is needed to assist HIV counsellors in convincing and rapidly preparing patients to start ART under the same-day ART initiation policy. There was a perception that patients who are uncertain about starting ART may require professional counselling, perhaps beyond the skill set of current lay counsellors. Adherence counselling was the broad term used by providers for the information session on ART. However, providers were uncertain of the exact content of the counselling provided, and opinion was divided on whether it was effective. Those who thought it was effective highlighted it as an essential facilitator in informing patients on clinic ART initiation procedures, psychological responses to HIV diagnosis ART and adherence expectations. Those in doubt of the effectiveness of adherence counselling referenced the high number of patient attrition and ART defaulters as an indication of its inadequacy (Table 4).

**Table 4:** Clinical management of HIV positive patients related facilitators and barriers to UTT policy and same-day ART initiation implementation

Barriers and facilitators	Illustrative quotes
Facilitators	
Knowledgeable patients and ART-ready patients	“Today we live with people who are HIV positive. Although we may speak of people who are in a remote place…and you know…this person…you may give us cues relating to that. But for somebody who is living in CBDs and all that urban life, and all that stuff. They should know about [HIV], even at workplace, I think that’s something…on TVs we talk about HIV…you know…or related things.”—PN “It doesn’t necessarily mean transmission will only be stopped by treatment. I think these days were just talking about treatment, treatment—we don’t talk about condoms anymore, we don’t talk about healthy lifestyle anymore. We just want treatment, treatment…”- PN
Reduced system barriers to ART	“… because like I say, the aim is to put everyone on treatment, everybody who is positive so that there must not be any transmission, and that’s how we deal with AIDS…”—PN
Barriers	
Counsellor skill limitations	“… we came up with this (policy), and now we are ready for UTT, but did we go back and look at the cadres of counselling that we have? To say, when they need to communicate that to the patients, how much intense can they go in order for the patients to be able to say “okay I can be motivated” [to initiate ART] or “no give me a chance [to think about initiating ART]”.”—PN “… I used to strongly believe that in issues where you do adherence counselling, there’s a certain length that the counsellor can go up to. Beyond that it needs a professional person.”—PN “We have got adherence counselling, but our non- suppressing patients, their levels [viral load] are very high. Which says to you that maybe the content or the counselling that is done is not getting through to the patients.”—PN
Limited patient ART demand creation efforts	“…people expected people will come in numbers to say, yah we want ARVs. But it’s not really happening like that because I think people are still exercising their right to choose whatever is that they want. Much as they said UTT…and then they thought the following day people will just come and say yah I want ARVs. It’s not…I don’t see it happening…”—PN “[Uptake of SDI]is very low. I think maybe it’s our mentality because some of the patients have been tested. They know the policy said “you will be initiated after the blood results”, but now all of a sudden something came up.”—PN
Patient and clinical readiness for ART	“No, I don’t think same-day ART initiation will work, the person has to accept that this is what is happening when he/she comes back from the clinic he/she shouldn’t be surprised… we will be able to assist him/her better than initiating him/her whilst still shocked, whilst crying. What is he/she going to do with the treatment? On the other hand, the husband tells her that he doesn’t want someone that takes a treatment. You see those kind of things? So that is no.”—LHC “… I think that it is fine if we allow a person to go and think about taking ARVs then if he/she has processed it and feels that he/she is ready that is when they can come back and say I am ready for starting on ARVs…”—LHC “Ask the patient to give you back the information that you have just told him/her, you will see that okay, and this person has heard what you said or he/she was not listening. That’s how I can tell that this one can initiate.”—LHC
Excess emphasis on ART targets, need to report ART deferral reasons	“now I’m doing my stats…and if I say my positivity rate…I tested twenty and my initiation rate for the week, this week, was 35%, I will be asked, why is it 35%. What do I say? It’s 35%, I initiated five out of the twenty and there’s nothing that I can do. It’s just a name-and-shame…and whatever reason you can come up with […] what you are saying, me I always write on the Treatment Retention Acceleration Program (TRAP) the reasons, but it doesn’t change the fact that I am at 35%, so I am pulling the region down. It’s not like these things we don’t say, and sometime if you talk, it’s like you are negative, you are not open to UTT, but those are the realities that we deal with at facility level, those are issues that are there at facility level and we need to talk about them”—PN

When HIV counsellors were asked about what their approach for addressing patient ambivalence with regards to starting ART, most lay HIV counsellors emphasised the dangers of not taking treatment and personal health benefits of taking up early ART. HIV counsellors may overly highlight the dangers of not starting ART, before resolving social barriers to adherence such as disclosure to partners and family. However, HIV counsellors who have had a positive personal experience with ART were more inclined to underscore the benefits of early ART. None of the HIV counsellors who were interviewed mentioned the benefit of ART as an HIV prevention tool.

“…we came up with this [policy], and now we are ready for UTT/SDI, but did we go back and look at the cadres of counselling that we have? To say, when they need to communicate that to the patients, how much intense can they go in order for the patients to be able to say “okay I can be motivated” [to initiate ART] or “no give me a chance [to think about initiating ART]”.”—PN.

“We have got adherence counselling, but our non- suppressing patients, their levels [viral load] are very high. Which says to you that maybe the content or the counselling that is done is not getting through to the patients.”—PN.

#### Limited patient ART demand creation efforts

Providers indicated that there was no systematic messaging or marketing regarding the new universal-test-and-treat and same-day ART policies. Thus, clinics reported low demand for same-day ART initiation, which was mainly provider-driven. Providers noted that patients may still remember past ART initiation procedures, including blood collection for baseline laboratory tests, a second visit (a week later) to receive blood test results and ART initiation later in the process. Additionally, primary care providers seemed uncertain about effective ways for engaging patients who were previously ineligible for ART [25], and forced to defer treatment initiation. There was mention of booking them for ongoing or adherence counselling. However, there seemed to be no systematic process for tracing previously ineligible HIV infected patients to now initiate them on ART.

“…people expected people will come in numbers to say, yah we want ARVs. But it’s not really happening like that because I think people are still exercising their right to choose whatever is that they want. Much as they said UTT…and then they thought the following day people will just come and say yah I want ARVs. It’s not…I don’t see it happening…”—PN.

#### Patient readiness for ART

Besides clinical readiness for same-day ART, many health providers were concerned that patients' social and emotional readiness for ART may be neglected. ART initiation on the day might be overwhelming and too sudden for some patients, implying that only highly motivated patients would take up ART on the day of HIV diagnosis and remain in care as required. There were concerns that rushing patients who need space to process the new diagnosis could result in disconnection from care after the initial acceptance of ART. Therefore, health providers favoured giving patients the necessary time to absorb the diagnosis and deal with the social prerequisites for sustainable ART adherence such as disclosure to a spouse/partner or family members, and arrangement for proper storage of antiretroviral drugs in their homes or workplaces. Health providers also stressed the importance of patients' right to choose whether/when they take up ART.

However, the process for assessing patient readiness was poorly defined among the different types of providers and clinics. Unless the patient verbally indicated that they were not ready to start treatment, assessment of treatment readiness seems to depend on provider observations and attitude to immediate ART. Some counsellors deemed patients to be ready for ART if they demonstrated understanding of the information shared during the counselling session, others mentioned patients' adherence to the post-HIV diagnosis follow-up visit schedule (i.e. returning a week later to collect baseline blood results) as an indication of readiness and commitment to lifelong ART.

“No, I don’t think same-day ART initiation will work, the person has to accept that this is what is happening when he/she comes back from the clinic he/she shouldn’t be surprised… we will be able to assist him/her better than initiating him/her whilst still shocked, whilst crying. What is he/she going to do with the treatment? On the other hand, the husband tells her that he doesn’t want someone that takes a treatment. You see those kinds of things? So that is no.”—LHC.

“Ask the patient to give you back the information that you have just told him/her, you will see that okay, and this person has heard what you said or he/she was not listening. That’s how I can tell that this one can initiate.”—LHC.

“I think again patients’ readiness, there’s subjective readiness and there’s objective readiness. My being readiness to [initiate] doesn’t necessarily meaning I qualify for you to initiate, we still have those patients that we will need to fast-track, the guidelines remains the same, antenatal, TB patients. I might be ready to take but then we have to exclude other conditions. You find out I’m ready, I’m saying yes, I’m ready, probably because… when you get deeper into literature, you know those patients, there’s certain categories of patients that you know such patients are better ready compared to these patients.:—PN.

#### Emphasis on ART targets, need to report ART deferral reasons

Healthcare managers expressed concerns that HIV monitoring indicators focused mainly on ART initiation numbers, excluding provider adherence to clinical guidelines and patient preferences that may affect longer-term patient outcomes. HIV monitoring tools do not include indicators to explain failures in same-day ART initiation implementation. These monitoring challenges created frustration and sometimes undue influence to initiate patients on ART even when they are not ready for it.

“now I’m doing my stats…and if I say my positivity rate…I tested twenty and my initiation rate for the week, this week, was 35%, I will be asked, why is it 35%. What do I say? It’s 35%, I initiated five out of the twenty and there’s nothing that I can do. It’s just a name-and-shame…and whatever reason you can come up with […] what you are saying, me I always write on the Treatment Retention Acceleration Program (TRAP) the reasons, but it doesn’t change the fact that I am at 35%, so I am pulling the region down. It’s not like these things we don’t say, and sometime if you talk, it’s like you are negative, you are not open to UTT, but those are the realities that we deal with at facility level, those are issues that are there at facility level and we need to talk about them”—PN.

## Discussions

South Africa has made a substantial investment in its commitment to universal treatment coverage by adopting the UTT and SDI guidelines and is committing to achieving the UNAIDS 95-95-95 targets by 2030 [2–4]. This is one of the first studies to gauge the perspectives and experiences of primary healthcare providers in the assimilation and implementation of the UTT and SDI policies in South Africa. This information is critical in understanding gaps in policy implementation process to improve the health systems' performance in South African and also other many LMICs that have adopted these policies particularly in the Sub-Saharan African context.

We found that primary care providers were knowledgeable about the UTT and SDI policies, and generally regarded them positively, highlighting the clinical and public health benefits. Similar opinions were shared by health care providers in high-income and other LMIC settings during ART eligibility expansion from a CD4 of 350–500 cells/µl, and after UTT policy adoption, and likewise among those implementing the Option B+ strategy of universal-testing and initiation of lifelong ART among all HIV-positive pregnant and breastfeeding women [26–31].

Study participants identified health system resource and capacity challenges in the implementation of the UTT and SDI policies in South Africa. Key barriers highlighted by providers were the surge in workload coupled with constrained healthcare infrastructure and human resources for health. Increases in workload were projected in modelling studies from before policy changes [7, 32]. An estimated 10.3 million HIV-positive individuals became eligible for ART in Sub-Saharan Africa when the UTT policy was adopted [7, 32–34]. In general, the existing infrastructure and health human resource challenges were not substantially addressed in LMICs [31, 35–38]. Adequate health resources are known to be critical components in ensuring quality health services and positive patient outcomes [28, 38–41]. While NGO partners have been instrumental in supporting policy implementation in South Africa and Sub-Saharan Africa [42, 43], they often cannot improve long-term infrastructural needs [42, 44–46]. In South Africa, government-led initiatives such as the Ideal Clinic Realisation and Maintenance (ICRM) programs and the Integrated Chronic Disease Management (ICDM) model are attempts to correct resource and infrastructure deficiencies and improve the quality of primary health care services [36, 47].

Similar to other studies, our findings also noted healthcare providers' concerns regarding patients feeling overwhelmed at the prospect of initiating lifelong ART immediately after diagnosis [28, 30, 31, 48, 49]. At the same time, study participants were uncertain of ways of assessing ART readiness challenges and were unclear of measures to ensure that patients who choose to defer ART remain engaged with the health system and promptly initiate ART when ready. Stated health-system and patient-related implementation challenges coupled with pressure on providers to meet ART initiation targets may inadvertently compromise considerations for patient-level factors to ART readiness [21, 49]. Patient readiness to start life-long ART is complex and motivated by many personal and social factors and has been established as an important determinant of ART uptake and future adherence to ART [18, 25, 50, 51]. Unprepared patients who are compelled to initiate ART may disengage from care, ultimately limiting the potential benefits of the SDI policy provision.

ART uptake has increased substantially since the adoption of the UTT policy in South Africa and other Sub-Saharan African countries [52–54]. However, recent evidence point to declining patient retention rates [55, 56]. Routine clinic data from Johannesburg and Mopani districts in South African districts showed a 45% higher likelihood of disengagement from care after six months from patients initiated on the same day of diagnosis compared to patients initiated later [56].

Patient counselling is essential to helping patients to accept their HIV positive status and prepare for life-long ART, particularly among patients who are diagnosed at a relatively healthy state and may not perceive the immediate benefits to early ART [28, 57–59]. Study participants noted challenges regarding current counsellors' capacity to manage patients ambivalence about immediate ART as well as non-compliant patients. The need for improved, quality counselling in the era of same-day ART initiation was noted among study participants, a factor which has been previously highlighted [28, 59–61].

Helpful strategies to overcome some of the policy implementation challenges include differentiated-service-delivery (DSD) ART models aimed at decongesting primary health clinics facilities and freeing up professional health worker time to focus on more complicated and sick patients [62–64]. Improved counselling strategies are needed to address patient ART readiness and improve long-term ART adherence and retention in care. Additionally, community-focused health promotion and media campaigns are needed to improve patient understanding of the benefits of early ART and their demand for same-day ART [28, 59, 65].

The findings from our study provide valuable insights from professionals at the forefront of ART policy implementation to further support South Africa's commitment to expanding access to ART. Policymakers will need to address the identified implementation challenges in collaboration with frontline implementers to maximise the demonstrated benefits of the UTT and SDI policies.

### Limitations

Limitations of the study include a small sample size. Data presented are from eleven clinics in the Gauteng province, which may be different from other facilities in the province and the country. Also, findings were based on opinions and perspectives of key informants who were health providers from a small subset of clinics in the Johannesburg metropolitan area, rather than on empirical data from clinics. Lastly, the qualitative design also limits the generalizability of these findings.

## Conclusions

Our results highlight important gaps in the drive to achieve the second UNAIDS 95% (diagnosed patients on ART) target. Specifically, the study demonstrates the need for further engagement with healthcare providers about the SDI policy, particularly infrastructural/capacity needs. Additionally, improved promotion of immediate ART in health care settings and media communication is needed to increase patient demand for early ART.

## Data Availability

Qualitative data extracts are presented in the article to support the findings. The original transcripts are not available to the public as they may contain information that could compromise the confidentiality of study participants.

## Abbreviations

ART: Antiretroviral therapy
CD4: Cluster of differentiation 4
DSD: Differentiated service delivery
FGD: Focus group discussion
HIV: Human immunodeficiency virus
HREC: Human Research Ethics Committee
KI: Key informant
LHC: Lay HIV counsellor
NGO: Non-governmental organisation
NIMART: Nurse initiated management of antiretroviral therapy
PHC: Primary health care
PN: Professional nurse
UNAIDS: Joint United Nations Programme on HIV/AIDS
WHO: World Health Organisation
